# PSEN1 is associated with colon cancer development *via* potential influences on PD-L1 nuclear translocation and tumor-immune interactions

**DOI:** 10.3389/fimmu.2022.927474

**Published:** 2022-08-17

**Authors:** Wangzhi Wei, Yu Zhang

**Affiliations:** Life Science Institute, Jinzhou Medical University, Jinzhou, China

**Keywords:** γ-secretase, presenilin 1 (PSEN1), PD-L1, colon cancer, tumor - immune interaction

## Abstract

Presenilin 1 (PSEN1), as a catalytical core of the γ-secretase complex, plays multiple actions through mediating transmembrane domain shedding of the substrates. Unlike extensive studies performed on investigating the functions of γ-secretase substrates or the effects of γ-secretase inhibitors, our findings uncover a potential action of PSEN1 on PD-L1 alternative truncation and nuclear translocation, broadening our understanding on how the γ-secretase contributes to colon cancer development as well as suggesting a potential strategy to improve the efficacy of PD-1/PD-L1 blockade. Immunohistochemical data showed loss of PD-L1 protein expression in all the primary colon adenocarcioma (COAD) cases in the HPA collection, while PSEN1 was scored to be highly expressed, indicating their converse expression patterns (p<0.001). Meanwhile a strongly positive gene correlation was explored by TIMER2 and GEPIA (p<0.001). Up-regulated PSEN1 expression in COAD might facilitate liberating a C-terminal PD-L1 truncation *via* proteolytic processing. Then following an established regulatory pathway of PD-L1 nuclear translocation, we found that PSEN1 showed significant correlations with multiple components in HDAC2-mediated deacetylation, clathrin-dependent endocytosis, vimentin-associated nucleocytoplasmic shuttling and importin family-mediated nuclear import. Moreover, connections of PSEN1 to the immune response genes transactivated by nuclear PD-L1 were tested. Additionally, contributions of PSEN1 to the tumor invasiveness (p<0.05) and the tumor infiltrating cell enrichments (p<0.001) were investigated by cBioportal and the ESTIMATE algorithm. Levels of PSEN1 were negatively correlated with infiltrating CD8+ T (p<0.05) and CD4+ T helper (Th) 1 cells (p<0.001), while positively correlated with regulatory T cells (Tregs) (p<0.001) and cancer associated fibroblasts (CAFs) (p<0.001). It also displayed significant associations with diverse immune metagenes characteristic of T cell exhaustion, Tregs and CAFs, indicating possible actions in immune escape. Despite still a preliminary stage of this study, we anticipate to deciphering a novel function of PSEN1, and supporting more researchers toward the elucidations of the mechanisms linking the γ-secretase to cancers, which has yet to be fully addressed.

## Introduction

Regulated intramembrane proteolysis (RIP) by γ-secretase is critical for a variety of physiological functions and cell biological processes (1, 2). The γ-secretase complex is comprised of four components including presenilins (PSEN1, PSEN2), nicastrin (NCSTN), presenilin enhancer (PSENEN) and anterior pharynx defective-1 homologs (APH1A or B) (3–5), and has emerged as a membrane-embedded protease with the ability to cleave transmembrane helices of type I protein substrates (6). Presenilins, the catalytical core of the complex, are subject to autoproteolytic cleavages yielding active N-terminal (NTF) and C-terminal fragments (CTF) (6), which play multiple actions through mediating transmembrane domain shedding of the substrates, leading to the release of the biologically active cytoplasmic domains of the substrate proteins, and subsequent translocation into the nucleus where they serve to regulate gene expression or interactions with cytosolic proteins to regulate their functions (6–8). As for other three co-factors, nicastrin is essential for substrate recognition, and facilitates presenilin- dependent proteolytic activities (7, 9). APH-1 homologs act as scaffolding proteins (7), and PSENEN appears to be an integral component to support the coordinated expression of presenilins and nicastrin (7, 10).

The γ-secretase complex has been documented to interact with a diverse range of substrates, of which the AD-linked amyloid precursor protein (APP) and Notch are intensively studied (6). The corresponding inhibitors of γ-secretase have been developed as drugs targeting Alzheimer’s disease and Notch-dependent tumors (6, 11). As numerous candidates have been identified and, continuously, more are being revealed, relevant studies are mainly focused on investigating the functions of the substrates or the effects of the inhibitors. However potential actions of the individual γ-secretase components, especially the proteolytically active presenilin 1 (PSEN1) subunit and the potential links between PSEN1 and caners have yet to be fully addressed. Recently, miR-193a, miR-654-3p and other selective PSEN1 inhibitions have been reported to hamper gastric (12), SNSCC (13) and Leukemias (14) cancer hallmarks, respectively. Thus, we explored PSEN1’s functional panorama, an interesting connection between PSEN1 and PD-L1 attracted our attention.

Membrane-anchored PD-L1 on tumor cells has been well recognized for its engagement with PD-1 on T cells to evade anti-tumor immunity (15). Upon protease -medicated domain shedding, active C-terminal PD-L1 fragment translocates from plasma membrane into nucleus (16). Accumulation of nuclear PD-L1 in tumor cells promotes the expression of multiple pro-inflammatory and immune response genes. Thus, strategies have been developed to improve the efficacy of PD-1/PD-L1 blockade by targeting PD-L1 nuclear localization (16). In addition, nuclear PD-L1 has been found to be upregulated in lung metastatic tumors, suggesting a potential interplay between tumor aggressiveness and PD-L1 translocation (17). Thus in-depth exploration of the regulatory mechanisms underlying PD-L1 nuclear translocation will improve better understanding of immune surveillance and tumor metastasis. Intrigued by the intrinsic immune suppressive microenvironment and high frequency of immune evasion of colon cancer (18, 19), we postulate that PSEN1 may contribute to the regulation of PD-1/PD-L1 signaling in colon cancer, and thus investigate the relationship between PSEN1 and PD-L1 translocation.

## Materials and methods

### Tumor immune estimation resource (TIMER) analyses

TIMER2.0 (http://timer.cistrome.org/) is used for comprehensive analyses of functions of infiltrating immune cells and molecular characteristics of tumor-immune interactions (20–22). The upgraded version integrates multiple algorithms for investigating various associations between immune infiltrates and genetic or clinical features in the TCGA cohorts, instead of only using one algorithm in the original TIMER (20).

Correlations between PSEN1 and the related gene markers were analyzed *via* the Gene Correlation module of the ‘Cancer Exploration’ component, based on 458 of primary colon adenocarcinoma (COAD) samples. The heat map tables of spearman’s correlation, as well as the corresponding scatter plots of the correlation between a pair of genes were generated, together with the partial rho coefficient. Using the same TCGA samples, correlations of PSEN1 with abundance of the immune infiltrates were explored *via* the Gene module of the ‘Immune Association’ component. The infiltration levels of CD4+ T cells, CD8+ T cells, regulatory T cells (Tregs) and cancer associated fibroblasts (CAFs) were evaluated through the six algorithms as described (20). The functional heat map tables and the corresponding scatter plots were output with the correlation coefficients and statistical significance. All the gene read counts were normalized in TPM after the purity adjustment.

### Queries of TCGA data *via* cBioportal and GEPIA web resources

The cBioPortal for Cancer Genomics provides integrative analysis of cancer genomics and clinical profiles (23, 24). 213 of primary colon adenocarcinoma samples were collected from the TCGA cohort (TCGA, Firehose Legacy). Levels of PSEN1 mRNA expression were compared between the groups in the presence (n=68) or absence (n=145) of lymphovascular invasion. The read counts were quantified in RSEM.

GEPIA (Gene Expression Profiling Interactive Analysis) tool integrates the TCGA profiles across cancer and healthy cohorts using the pairwise gene correlation analysis (25). Correlations of PSEN1 with the related gene markers were analyzed based on the primary colon adenocarcinoma (n=275) and normal colon tissue (n=41) samples. The scatter plots were output with the Spearman's correlation coefficients and corresponding significance.

### The human protein atlas (HPA) and immunohistochemistry (IHC)

The Human Protein Atlas project creates a comprehensive overview of protein expression in human tissues (26–28). The expression of PSEN1 and PD-L1 (CD274) was screened across 20 types of the malignancies in the HPA. The protein levels were assessed by immunostaining using the PSEN1 HPA030760 antibody (Sigma #HPA030760, RRID : AB_10696211) or the CD274 CAB076385 antibody (CST #13684, RRID : AB_2687655), respectively. The color-coded bars indicate the percentages of the patients with high/medium PSEN1 or PD-L1 protein levels. Those with undetectable levels are shown in white bars. All the corresponding immunohistochemical images of the primary colon adenocarcinoma samples were collected, 9 of the cases stained with PSEN1 and 8 of those with PD-L1. In addition, formalin-fixed paraffin-embedded sequential colon adenocarcinoma sections were obtained from Outdo Biotech (Shanghai, China), stained with 1: 100 diluted anti-Presenilin 1 (Abcam#ab76083) or 1: 200 diluted CD274 (CST#13684) antibody. The images were visualized and exported by using the Case viewer 2.4 (3D HISTECH LTD) software. The evaluation of the PSEN1 and PD-L1 expression levels was based on the annotation parameters including staining intensity and fraction of stained cells. The staining intensity was classified into four levels, namely 0, 1+, 2+, and 3+, which were designated as negative, weakly positive, moderately positive, and strongly positive signals, respectively; The percentage of the stained cells was categorized as 0 (0%), 1(< 25%), 2 (25–75%) and 3 (>75%), respectively. The total score was calculated by multiplying the intensity score by the corresponding distribution score. A total score of six was used as a cutoff to compare the groups in high and low expression.

### Clinical proteomic tumor analysis consortium (CPTAC) *via* UALCAN

The clinicopathologic features of PSEN1 on colon cancer were evaluated by UALCAN, an interactive web portal to perform analyses using the cancer genomic, transcriptomic and proteomic data from several major cancer projects including TCGA and the Clinical Proteomic Tumor Analysis Consortium (CPTAC) (29–31). 97 of primary colon cancer and 100 of normal colon tissue samples were collected from the CPTAC and explored for PSEN1 *via* UALCAN. The protein expression levels were log2 normalized in Z-values. The individual cancer stages were categorized into Stage I (T1 or T2 N0 M0), Stage II (T3 or T4 N0 M0), Stage III (Tx N1 or N2 M0) and Stage IV(Tx Nx M1) (29)

### Estimation of infiltrating stromal and immune cells by ESTIMATE algorithm

ESTIMATE (Estimation of STromal and Immune cells in MAlignant Tumor tissues using Expression data) is used to calculate stromal and immune enrichment scores (32). The PSEN1 microarray data were collected from the primary colon adenocarcinoma samples in the TCGA cohort **(**TCGA, Firehose Legacy) *via* cBioportal. The corresponding stromal scores, immune scores and combined ESTIMATE scores were profiled from the Agilent G4502A microarray platform *via* ESTIMATE. Then the scores were compared between the dichotomized groups based on PSEN1 expression.

### Statistical analyses

**Figure 1:**
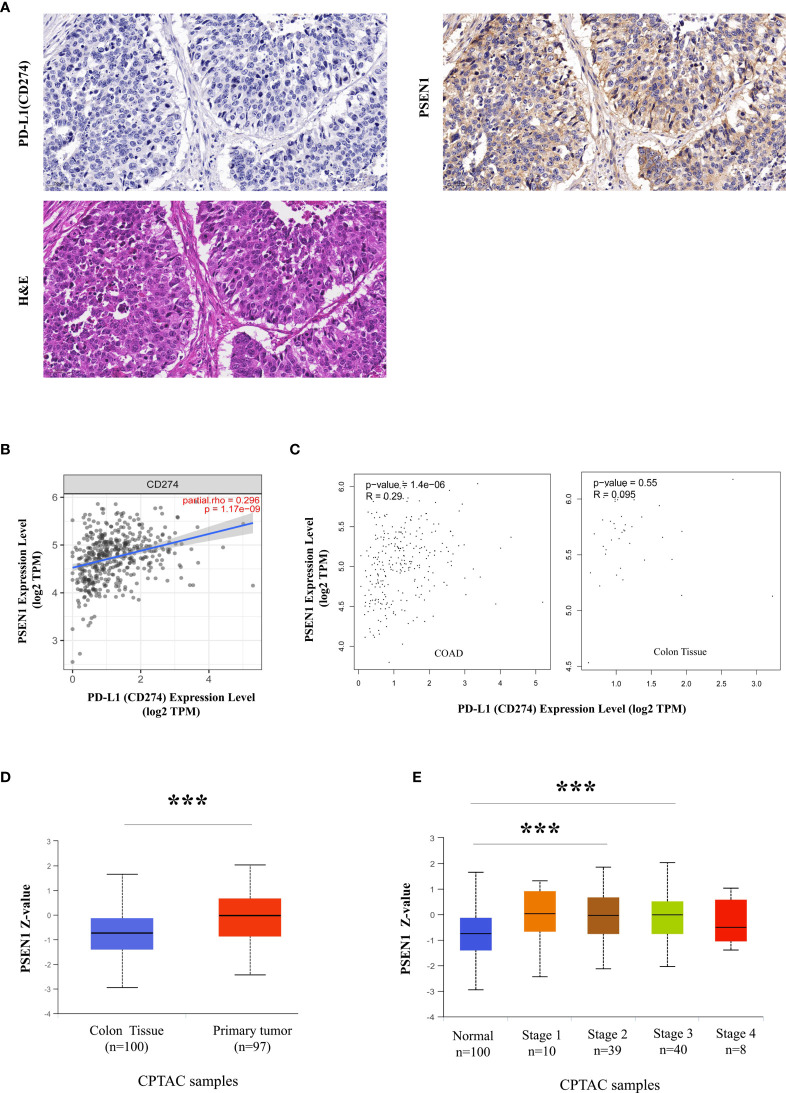
A potential protolytic cleavage of PD-L1 engaged by PSEN1. **(A)** Immunohistochemical images of PD-L1 (CD274) (upper left) and PSEN1 (upper right) expression on human primary colon adenocarcinoma (COAD) as well as the corresponding H&E staining (lower). A zoom scale =50μm. **(B)** The correlation between PSEN1 and PD-L1 (CD274) in COAD examined by TIMER2. **(C)** The correlations between PSEN1 and PD-L1 (CD274) in COAD samples as well as normal colon tissues explored by GEPIA. Levels of the mRNA expression were normalized in Log2 TPM, and the corresponding correlation coefficients and significance were indicated. **(D)** PSEN1 protein expression levels analyzed by UALCAN, box plots showing the levels of PSEN1 in normal (n = 100) and primary COAD samples (n = 97). **(E)** The protein levels of PSEN1 against colon cancer stages analyzed by UALCAN. Stage I: T1 or T2 N0 M0; Stage II – T3 or T4 N0 M0; Stage III – Tx N1 or N2 M0; Stage IV – Tx Nx M1. The protein expression levels were log2 normalized in Z-values, and the significance was indicated with the symbol (*** P < 0.001).

**Figure 2:**
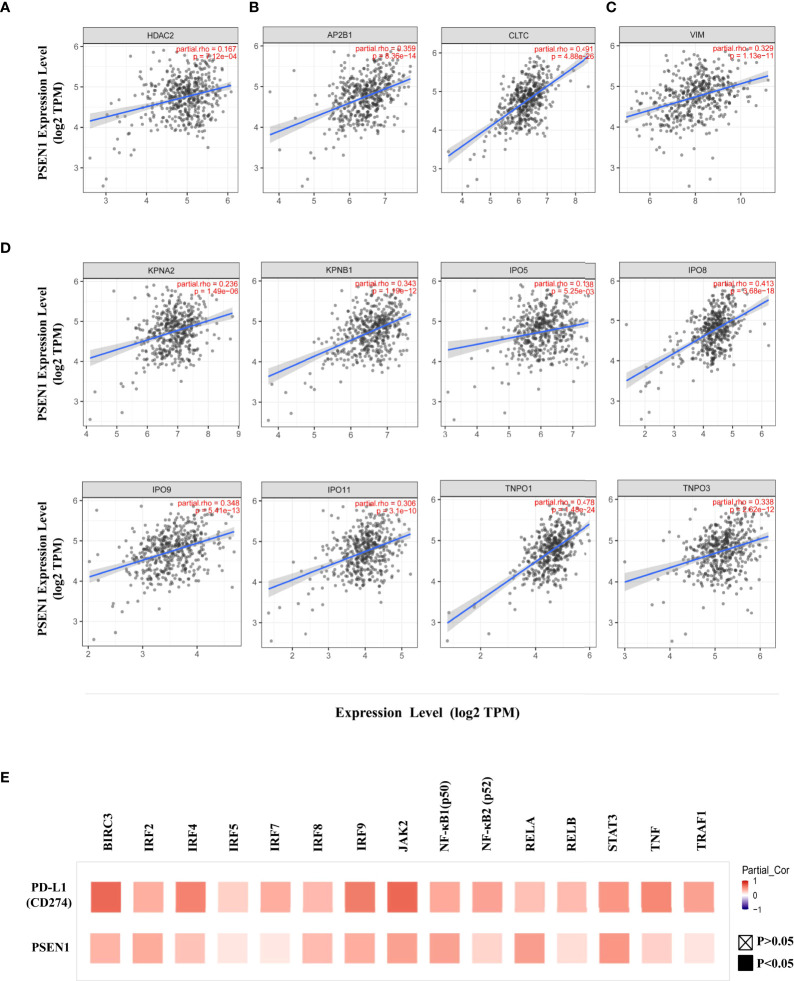
PSEN1 correlates with PD-L1 nuclear translocation in colon cancer. The correlations of PSEN1 with **(A)** HDAC2, **(B)** AP2B1 and clathrin (CLTC), **(C)** vimentin (VIM), **(D)** importin α1(KPNA2), importin β1 (KPNB1), importin 5 (IPO5), importin 8 (IPO8), importin 9 (IPO9), importin 11 (IPO11), transportin 1 (TNPO1) and transportin 3 (TNPO3) in COAD analyzed by TIMER2. Levels of the mRNA expression were normalized in Log2 TPM and the corresponding correlation coefficients and significance were indicated. **(E)** The correlations of PD-L1 (upper) or PSEN1 (lower) with the immune response genes in COAD analyzed by TIMER2 and shown in a heat map table of spearman’s correlation. Red indicates a statistically significant positive association, and blue indicates a statistically significant negative association. Gray denotes a non-significant result. P < 0.05, shown in solid squares and P > 0.05, shown in hollow squares.

All the comparisons were determined by T-test in **Figures 1D, E** and **Figures 4A, B**; In **Table 1**, Chi-square and Fisher’s exact tests were used for the staining analysis; Spearman’s correlation analyses were performed in **Figures 1B, C**, **2**, **4C** and **Tables 2** **–** **4**. Significance was shown as *P < 0.05, **P < 0.01, ***P < 0.001.

**Table 1:** Immunohistochemical Scores of PSEN1 and PD-L1 (CD274) in COAD.

	Colon Adenocarcinoma (COAD)	
	PSEN1	PD-L1
High Expression (Score>=6)	9	0
Low Expression (Score<6)	0	8

**Table 2:** Correlations in PD-L1 Nuclear Translocation.

Colon Adenocarcinoma (COAD)									
	Gene Marker	PSEN1 Correlation (TIMER 2.0)			Gene Marker	PSEN1 Correlation (TIMER 2.0)		PD-L1 Correlation (TIMER 2.0)	
		Purity-adjusted partial	P Value			Purity-adjusted partial	P Value	Purity-adjusted partial	P Value
		spearman's rho				spearman's rho		spearman's rho	
**PD-L1 Deacetylation**	HDAC2	0.167	***	**Nuclear PD-L1 regulated genes**	NFκB1 (p50)	0.42	***	0.387	***
					NFκB2 (p52)	0.203	***	0.412	***
**Endocytosis of PD-L1**	Clathrin	0.491	***						
	Adaptin β2 (AP2B1)	0.359	***		RELA (p65)	0.437	***	0.287	***
					RELB	0.162	**	0.309	***
					STAT3	0.469	***	0.467	***
**Cytoskeleton**	Vimentin	0.329	***						
					JAK2	0.421	***	0.658	***
**Nuclear Import **	Importin α1	0.236	***		TNFα	0.217	***	0.526	***
	Importin β1	0.343	***		TRAF1	0.14	**	0.419	***
	Importin 5	0.138	**		BIRC3	0.341	***	0.656	***
	Importin 8	0.413	***		IRF2	0.382	***	0.364	***
	Importin 9	0.348	***		IRF4	0.278	***	0.547	***
	Importin 11	0.306	***		IRF5	0.13	**	0.219	***
	Transportin 1	0.478	***		IRF7	0.121	*	0.369	***
	Transportin 3	0.338	***		IRF8	0.313	***	0.319	***
					IRF9	0.368	***	0.574	***

*P < 0.05, **P < 0.01, ***P < 0.001.

**Table 3:** Correlations between PSEN1 and the Gene Markers of the Immune Subsets determined by TIMER2.0.

Cell Type	Colon Adenocarcinoma (COAD)		
	Gene Marker	PSEN1 Correlation (TIMER 2.0)	
		Purity-adjusted partial	P Value
		spearman's rho	
T cell exhaustion	PD-1(PDCD1)	0.147	**
	CTLA4	0.228	***
	LAG3	0.189	***
	TIM-3 (HAVCR2)	0.35	***
Regulatory T cells	FOXP3	0.317	***
	TGFβ (TGFB1)	0.327	***
	IL-10	0.283	***
	PTGIR	0.163	**
	ITGA4	0.452	***
	STAT5B	0.328	***
CAFs	CD44	0.255	***
	α-SMA	0.215	***
	FAP	0.254	***
	FSP1 (S100A4)	0.141	**
	PDGFRα	0.394	***
	PDGFRβ	0.294	***

**P < 0.01, ***P < 0.001.

**Table 4:** Correlations between PSEN1 and the Gene Markers of the Immune Subsets determined by GEPIA.

Cell Type	Gene Marker	Colon Adenocarcinoma (COAD)		Colon Tissue	
		PSEN1 Correlation (GEPIA)		PSEN1 Correlation (GEPIA)	
		Correlation	*P* Value	Correlation	*P* Value
		spearman's R value		spearman's R value	
					
T cell exhaustion	PD-1(PDCD1)	0.22	***	-0.098	0.54
	CTLA4	0.28	***	-0.24	0.13
	LAG3	0.23	***	0.049	0.76
	TIM-3 (HAVCR2)	0.38	***	-0.25	0.12
Regulatory T cells	FOXP3	0.38	***	-0.15	0.34
	TGFβ (TGFB1)	0.37	***	-0.36	*
	IL-10	0.25	***	-0.46	**
	PTGIR	0.26	***	-0.43	**
	ITGA4	0.45	***	0.18	0.26
	STAT5B	0.29	***	-0.51	***
CAFs	CD44	0.23	***	-0.48	**
	α-SMA	0.28	***	-0.52	***
	FAP	0.2	***	-0.6	***
	FSP1 (S100A4)	0.12	*	-0.79	***
	PDGFRα	0.38	***	-0.36	*
	PDGFRβ	0.36	***	-0.3	0.06

*P < 0.05, **P < 0.01, ***P < 0.001.

## Results

### A potential proteolytic cleavage of PD-L1 engaged by PSEN1

Discordance between PD-L1 staining and clinical responses to anti-PD-L1 therapy challenges clinicians (17). Most cancer subtypes demonstrate substantial variations in PD-L1 protein- to- mRNA ratios, probably due to post-translational PD-L1 down-regulation (33). Loss of PD-L1 protein level in tumor cells is considered to be a result of its ectodomain shedding, producing a soluble form (33). Here as expected, screening of PD-L1 protein expression across 20 types of the malignancies in the Human Protein Atlas (HPA) showed a landscape of low frequency of PD-L1 positive staining (**Supplementary Figure 1A**). Among these cancer samples with low or undetectable PD-L1 levels, most cases were strongly or moderately positive in PSEN1 expression (**Supplementary Figure 1B**). As known that, a multiplicity of γ-secretase substrates have type I transmembrane protein domain structures, while PD-L1 is exactly featured with the structural characteristics of this class of proteins, which aroused our interests to further investigate their relationship in tumors. As the top ranking in PSEN1 expression (**Supplementary Figure 1B**), colon cancer was thus focused for our in-depth studies. Loss of PD-L1 protein expression was confirmed by IHC, based on all the primary COAD cases in the HPA collection (**Supplementary Figure 2A**). Conversely, by taking into consideration of the staining intensity and the percentage of stained cells, PSEN1 was scored to be highly expressed in all the COAD samples (**Supplementary Figure 2B**). To further assess this unique expression pattern, we re-stained PD-L1 and PSEN1 in sequential COAD sections and found that the expressions of PD-L1 and PSEN1 were mutually exclusive. Consistent with the HPA data, samples positive for PSEN1 were negative for PD-L1 (**Figure 1A**). A negative association at the protein levels was identified by Chi-square and Fisher’s exact test (p<0.001) (**Table 1**). However, a strongly positive gene correlation between PSEN1 and PD-L1 was verified by TIMER2 and GEPIA (p<0.001) (**Figures 1B, C**), and notably, this connection was tumor specific (**Figure 1C**). In such a scenario, considering low PD-L1 protein- to-mRNA ratios are indicative to predict unfavorable outcome (33), we thus evaluated the clinicopathologic features of PSEN1. UALCAN analysis on the CPTAC cohorts confirmed the up-regulation of PSEN1 in the primary COAD samples (n=97) when compared with the normal tissues (n = 100) (p < 0.001) (**Figure 1D**). Furthermore we interrogated the protein levels of PSEN1 against colon cancer stages. As shown in **Figure 1E**, PSEN1 was preferentially expressed on the early onset of colon tumorigenesis instead of the progressive stages.

Due to a lack of explicit documentations about substrate consensus sequences that can be specifically recognized by γ-secretase, elucidations on how γ-secretase recognizes its substrate remain elusive (11). Here, we would be more focused on how such a potential proteolytical processing engaged by PSEN1 may alter PD-L1 intracellular actions.

### PSEN1 correlates with PD-L1 nuclear translocation in colon cancer

Given that an acetylation-dependent regulation of PD-L1 nuclear localization has been established (16), we thus investigated the relationship between PSEN1 and various components of these pathways to discern how PSEN1 may affect PD-L1 nuclear translocation. Protein acetylation on lysine affects protein stability and subcellular localization (16, 34), while deacetylation on cytoplasmic K263 residue by histone deacetylase 2 (HDAC2) triggers PD-L1 nuclear translocation (16). Interestingly, we found that the expression of PSEN1 was positively correlated with that of HDAC2 in COAD (partial rho =0.167, p < 0.001) (**Figure 2A** **).** Coordinated HDAC2 protein up-regulation was also observed in the COAD samples (data not shown).

PD-L1 intracellular trafficking is initiated through clathrin-dependent endocytosis (16). In this process, clathrin (CLTC) selectively binds with cargo adaptors, in particularly *via* Adaptin β2 (AP2B1) to tether unacetylated PD-L1 to clathrin (16, 35). Based on this scenario, we explored the potential correlations of PSEN1 with AP2B1 or clathrin, and found that PSEN1 was significantly correlated with AP2B1 (partial rho = 0.359, p < 0.001) and clathrin (partial rho = 0.491, p < 0.001) (**Figure 2B** **).** Furthermore, the subsequent nucleocytoplasmic shuttling of PD-L1 is dependent on interactions with several cytoskeletal proteins, such as vimentin (VIM) in particular (16, 36). As expected, a significant association of PSEN1 with vimentin was observed in COAD (partial rho = 0.329, p < 0.001) (**Figure 2C**). Previous reports indicate that, as PD-L1 interacting proteins (16), some importin family members are also involved in regulating PD-L1 nuclear imports (16, 37). We therefore investigated the potential relationship of PSEN1 with these importins and transportins. As shown in **Figure 2D**, we found significant correlations between PSEN1 and various importin family members including importin α1(KPNA2) (partial rho = 0.236, p < 0.001), importin β1 (KPNB1) (partial rho = 0.343, p < 0.001), importin 5 (IPO5) (partial rho = 0.138, p < 0.01), importin 8 (IPO8) (partial rho = 0.413, p < 0.001), importin 9 (IPO9) (partial rho = 0.348, p < 0.001), importin 11 (IPO11) (partial rho = 0.306, p < 0.001), transportin 1 (TNPO1) (partial rho = 0.478, p < 0.001) and transportin 3 (TNPO3) (partial rho = 0.338, p < 0.001).

Nuclear PD-L1 has been shown to transactivate a panel of pro-inflammatory and immune response-related genes involved in interferon (IFN) signaling, NF-κB signaling and MHC I pathways and others, which are critical to control the responses to PD-L1/PD-1 blockade (16, 38), and we therefore performed a screening of these gene representatives. In addition to verify their established relationships to PD-L1, multiple correlation analyses of these genes with PSEN1 were further performed (**Figure 2E** and **Table 2**), including NF-κB p65 (RELA) (partial rho = 0.437, p < 0.001), RELB (partial rho = 0.162, p < 0.01), NF-κB1 (p50) (partial rho = 0.42, p < 0.001), NF-κB2 (p52) (partial rho = 0.203, p < 0.001), JAK2 (partial rho = 0.421, p < 0.001), STAT3 (partial rho = 0.469, p < 0.001), TNF Receptor Associated Factor 1 (TRAF1) (partial rho = 0.14, p < 0.01), BIRC3 (partial rho = 0.341, p < 0.001), TNFα (partial rho = 0.217, p < 0.001), Interferon Regulatory Factor 2 (IRF2) (partial rho = 0.382, p < 0.001), IRF4 (partial rho = 0.278, p < 0.001), IRF5 (partial rho = 0.13, p < 0.01), IRF7 (partial rho = 0.121, p < 0.05), IRF8 (partial rho = 0.313, p < 0.001) and IRF9 (partial rho = 0.368, p < 0.001). These significant positive interactions of nuclear PD-L1 transactivating target genes were indicative that PSEN1-engaged events might influence immune response genes through the PD-L1 nuclear binding motifs.

**Figure 3:**
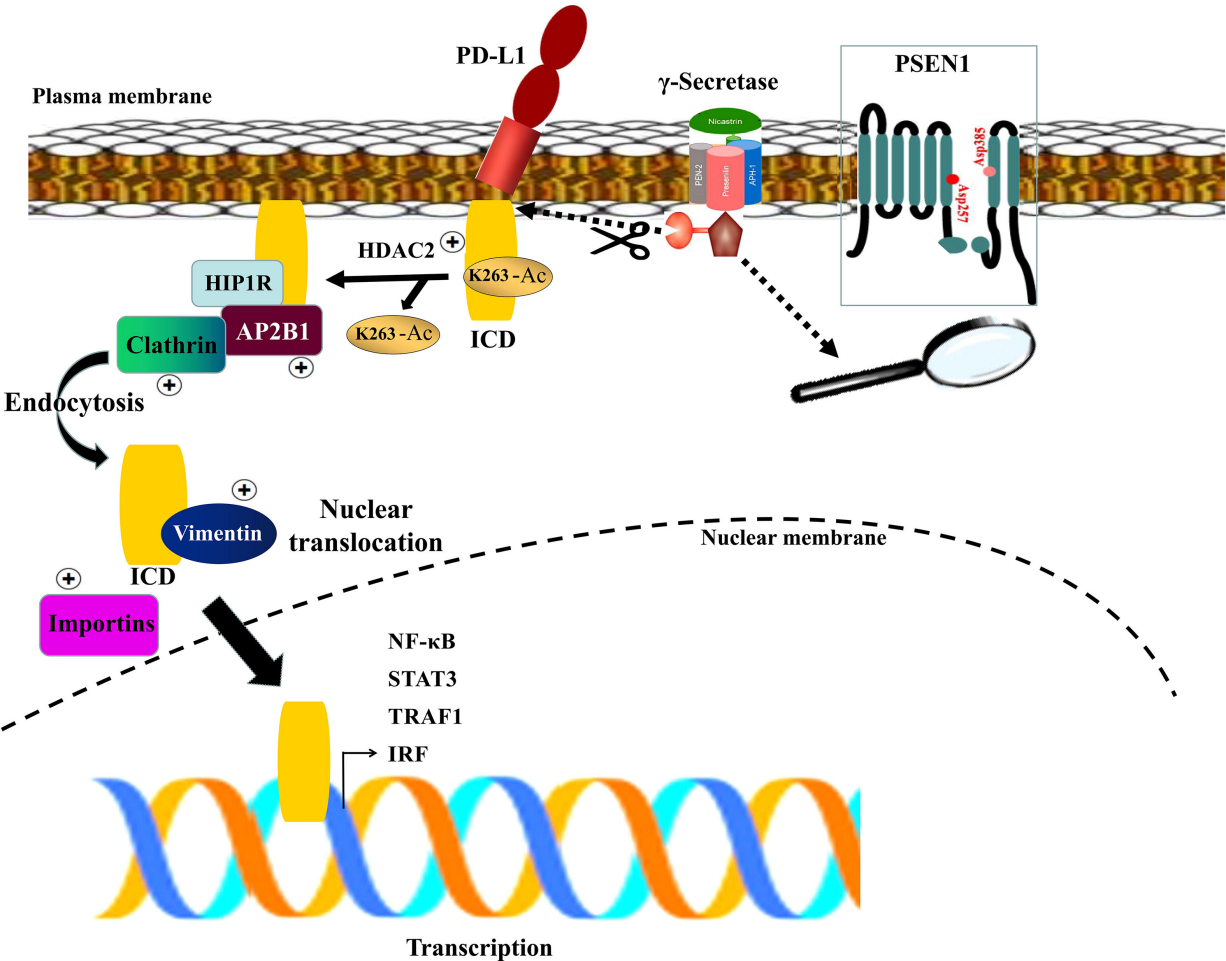
A schematic diagram of PSEN1-engaged PD-L1 nuclear translocation. The γ-secretase complex is comprised of four components with Presenilin (PSEN1) as the catalytic core containing two catalytically active aspartates (Asp257 and Asp385) located in the NTF and CTF. Upon assembly with the other subunits, PSEN1 undergoes autoproteolysis resulting in the active complex which might facilitate PD-L1 truncation *via* potential proteolytic processing, liberating its active C-terminal fragment (ICD), and induce PD-L1 nuclear translocation through HDAC2-mediated deacetylation, clathrin-dependent endocytosis, vimentin-associated nucleocytoplasmic shuttling and importins-mediated nuclear import. Subsequently nuclear PD-L1 transactivates a panel of pro-inflammatory and immune response -related genes.

In the light of our findings and the supporting documentations (16, 39–41), we propose a model for PSEN1 to regulate tumor-immune interactions *via* influencing PD-L1 nuclear translocation (**Figure 3**). In this model, the upregulated PSEN1 in colon cancer facilitates PD-L1 truncation *via* potential proteolytic processing, releasing its active C-terminal fragment, and then induces PD-L1 nuclear translocation through impacts on the multiple components in HDAC2-mediated deacetylation, clathrin-dependent endocytosis, vimentin-associated nucleocytoplasmic shuttling and importin family-mediated nuclear import. Nuclear PD-L1 subsequently transactivates a panel of pro-inflammatory and immune response-related genes, building up a positive feedback loop of increased nuclear PD-L1 expression to foster the evasion of immune surveillance.

### PSEN1 impacts invasiveness and immunophenotypes in colon cancer

**Figure 4:**
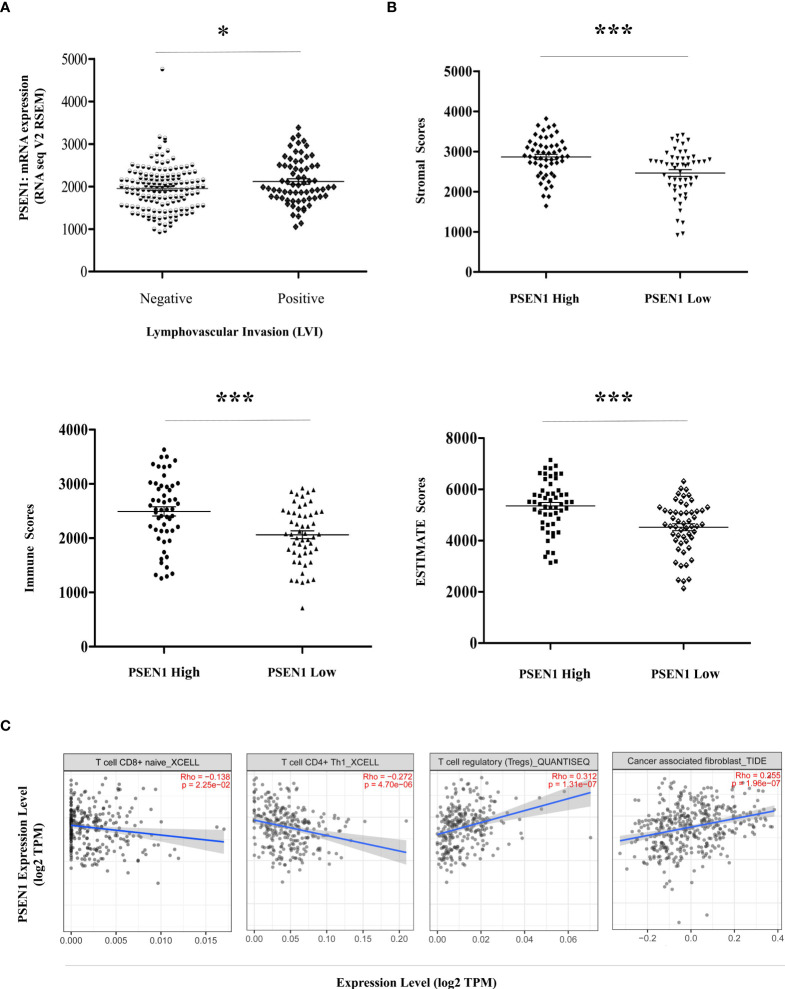
PSEN1 impacts invasiveness and immunophenotypes in Colon Cancer. **(A)** Levels of PSEN1 mRNA expression compared between the COAD cases in the presence (n=68) or absence (n=145) of lymphovascular invasion. The read counts were quantified in RSEM. **(B)** The stromal scores (upper), immune scores (lower left) and combined ESTIMATE scores (lower right) compared between the dichotomized COAD cases based on PSEN1 mRNA expression. The significance was indicated with the symbols (*P<0.05 ***P<0.001). **(C)** Correlations of PSEN1 with the infiltration abundance of naïve CD8^+^ T cells, CD4^+^ Th1 cells, Tregs and cancer associated fibroblasts (CAFs) explored by TIMER2.0. The purity-adjusted spearman’s rho and significance were indicated.

Given the potential interaction of PSEN1 with the cytoskeleton protein vimentin (**Figure 2C**) which is involved in mediating epithelial- to- mesenchymal transition (EMT) and metastasis (16), we thus interrogated the involvement of PSEN1 in tumor invasiveness. cBioportal analysis demonstrated significant upregulation of PSEN1 in COAD patients with lymphovascular invasion (LVI) (p< 0.05) (**Figure 4A**). As stromal and immune signatures in TME are predictive of tumor invasiveness and metastasis, we next evaluated the contributions of PSEN1 to the tumor infiltrating stromal and immune cell enrichments as shown **Figure 4B** (p<0.001). To further uncover the cellular composition of the infiltrates affected by PSEN1 in COAD, we performed correlation analyses of PSEN1 with individual immune subsets and found that levels of PSEN1 were negatively correlated with naive CD8^+^ T (Spearman’s rho = −0.138, p < 0.05) and CD4^+^ Th1 cells (Spearman’s rho = −0.272, p < 0.001), but positively correlated with Tregs (Spearman’s rho = 0.312, p < 0.001) and CAFs (Spearman’s rho = 0.255, p < 0.001) (**Figure 4C**). Furthermore, we evaluated the relationship between PSEN1 and a compendium of gene representatives characteristic of T cell exhaustion, Tregs and CAFs. As summarized in **Table 3**, PSEN1 displayed significant associations with these immune metagenes in COAD, and the interactions were further confirmed by GEPIA (**Table 4**). Intriguingly, none of the significant correlations described above was observed in normal colon tissues (**Table 4**), suggesting that these interactive events might be specifically occurred in tumor microenvironment. Together, these data support potential actions of PSEN1 in tumor - immune interactions, which might confer an alternate escaping mode of PD-L1 against the host immunity.

## Discussion

In this study, we demonstrate that PSEN1 may contribute to the regulation of PD-1/PD-L1 signaling through potential proteolytic processing of PD-L1, supporting PD-L1 nuclear accumulation. In order to achieve a comprehensive collection of patient cases, we took advantage of vast numbers of open-source data from the major cancer projects such as TCGA and CPTAC for exploration of the interactive expression patterns between PSEN1 and PD-L1 as well as the multiple correlations of PSEN1 with PD-L1 nuclear translocation, which overcame the obstacle of our own limited sample collection.

A multiplicity of γ-secretase substrates possess type I transmembrane protein domain structures, while PD-L1 is exactly featured with the structural characteristics of this class of proteins. The presence of a short ectodomain might be a critical structural priority of PD-L1 as a potential substrate of γ-secretase, which is typically achieved by the ectodomain shedding mediated by the ADAM families (42). Such a structural feature in γ-secretase substrates is mainly to adapt to subsequent nicastrin binding and substrate recognition (6). Following initial interactions with the complex, the substrates are recruited to the γ-secretase active cleavage site which is composed of two catalytically active aspartates (Asp257 and Asp385) located in the NTF and CTF of PSEN1 (6). The γ-secretase cleavage events are recognized to be carried through a sequential manner including canonical cleavages near the middle of the substrate transmembrane domain (TMD) followed by a second ϵ -site cleavage occurred close to the C-terminal limit of the TMD, leading to liberating the non-membrane anchored fragments (43). However, due to a lack of explicit documentations about substrate consensus sequences that can be specifically recognized by the γ-secretase complex, exactly which properties of the TMD are sufficient to define PD-L1 as a γ-secretase substrate still need to be further determined. But this does not hinder us from investigating how such a potential proteolytical cleavage engaged by the γ-secretase may alter PD-L1 intracellular actions.

In the light of our findings, we propose such a model that upregulated PSEN1 in colon cancer might facilitate PD-L1 truncation *via* potential proteolytic processing, releasing its active C-terminal fragment which is subject to HDAC2-mediated deacetylation and subsequent nuclear translocation. Consequently, increased nuclear PD-L1 in tumor cells promotes the expression of multiple pro-inflammatory and immune response genes, fostering potential evasion of immune surveillance. In addition, nuclear PD-L1 may induce acquired immunotherapy resistance by transactivating other immune checkpoint molecules that are not targeted by the PD-1/PD-L1 blockade, such as VISTA and B7-H3 (16). Thus targeting PD-L1 nuclear localization might augment anti-tumor immune responses. Here, our studies provide a possible rationale for combining PSEN1 inhibition with PD-1/PD-L1 blockade as a potential immunotherapy for colon cancer.

Given the potential interplay between tumor invasiveness and PD-L1 translocation, we unmask a landscape of tumor infiltrating lymphocytes (TILs) in TME engaged by PSEN1. The stepwise tumor immunity events are eventually executed through CD8+ T cell‐directed tumor cytolysis, targeting cancer cells by granule exocytosis and Fas ligand (Fas L)‐mediated apoptosis as well as IFN‐γ or TNFα -induced cytotoxicity (44). The abundance of cytotoxic CD8+ T lymphocytes infiltrated to the tumor invasive margins is predictive of beneficial clinical outcomes, influencing the efficacy of immune checkpoint blockades (44). CD4+ Th1 cells amplify cytotoxic T lymphocyte responses by assisting their priming relayed through specific DCs (45), and target tumor cells either directly through cytolytic mechanisms or indirectly by modulating the tumor microenvironment (45). Whereas a high infiltration of inhibitory regulatory T cells, especially Foxp3^high^ Tregs, promotes immunosuppression in the TME and associates with a poor cancer prognosis (18). CAFs attenuate CD8+ T cell activity, and by secreting various growth factors, cytokines, and chemokines, CAFs stimulate tumor cell transformation and facilitate cell proliferation, metastasis, angiogenesis as well as drug resistance, which in turn shield tumor cells from immune surveillance (18). The significant correlations of PSEN1 with these major immune subsets as well as the associated gene representatives suggest possible roles of PSEN1 in immune escape.

## Data availability statement

The datasets presented in this study are available in online repositories. These data were derived from the following publicly available resources:

1. TIMER2: http://timer.cistrome.org
2. cBioportal: https://www.cbioportal.org
3. GEPIA: http://gepia.cancer-pku.cn
4. UALCAN: ualcan.path.uab.edu
5. The Human Protein Atlas (HPA): https://www.proteinatlas.org
6. ESTIMATE: https://bioinformatics.mdanderson.org/estimate

## Author contributions

WW: study design, acquisition of data and manuscript writing; YZ: general guidance and supports. All authors contributed to the article and approved the submitted version.

## Funding

This work was supported by the Chongqing International Institute for Immunology (#2020YJC04).

## Conflict of interest

The authors declare that the research was conducted in the absence of any commercial or financial relationships that could be construed as a potential conflict of interest.

## Publisher’s note

All claims expressed in this article are solely those of the authors and do not necessarily represent those of their affiliated organizations, or those of the publisher, the editors and the reviewers. Any product that may be evaluated in this article, or claim that may be made by its manufacturer, is not guaranteed or endorsed by the publisher.

## Supplementary material

Supplementary Figure 1 Protein expression summary across cancers in the Human Protein Atlas (HPA) **(A)** PD-L1 (CD274) and (B) PSEN1 protein expressions in different type of TCGA tumors were summarized in the HPA. The color-coded bars indicate the percentages of the patients with high/medium PD-L1 or PSEN1 protein levels. Those with undetectable levels are shown in white bars.

Supplementary Figure 2 Immunohistochemical images of all the primary COAD samples in the HPA collection, 8 of the cases stained with PD-L1 (CD274) **(A)** and 9 of those for PSEN1 **(B)**. The evaluation of the PD-L1 and PSEN1 expression levels was based on the annotation parameters (upper). The staining intensity is classified into four levels, namely 0, 1+, 2+, and 3+, which are designated as negative, weakly positive, moderately positive, and strongly positive signals, respectively; The percentage of the stained cells is categorized as 0 (0%), 1(< 25%), 2 (25–75%) and 3 (>75%), respectively.

Supplementary Figure 3 Correlations in PD-L1 nuclear translocation The correlations of the components involved in PD-L1 nuclear translocation in COAD analyzed by TIMER2 and shown in a heat map table of spearman’s correlation. Red indicates a statistically significant positive association, and blue indicates a statistically significant negative association. Gray denotes a non-significant result. P < 0.05, shown in solid squares and P > 0.05, shown in hollow squares.
