# Supplementary material for: PSEN1 is associated with colon cancer development via potential influences on PD-L1 nuclear translocation and tumor-immune interactions
Source: Front Immunol. 2022 Aug 17;13:927474. doi: 10.3389/fimmu.2022.927474 (PMC9428321; doi:10.3389/fimmu.2022.927474)

**Supplementary Figure 1**

**A**

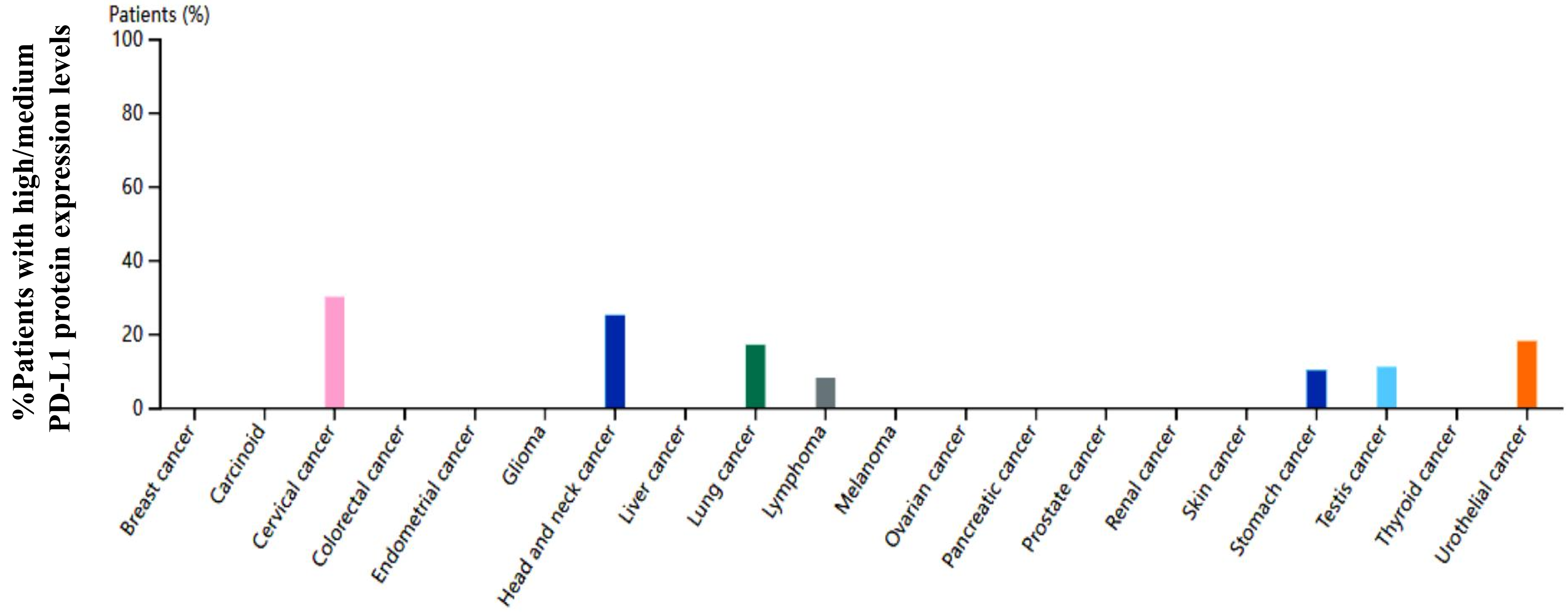


**B**

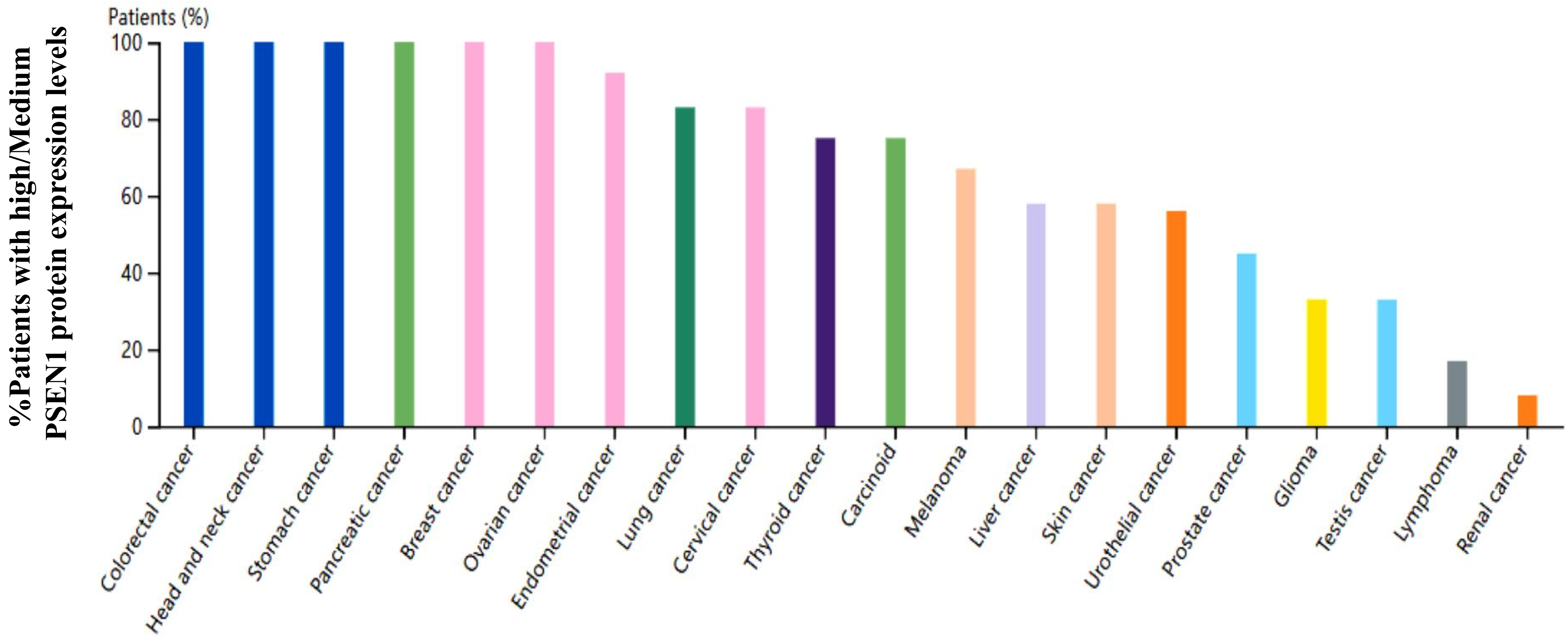

**Supplementary Figure 2**

**A**

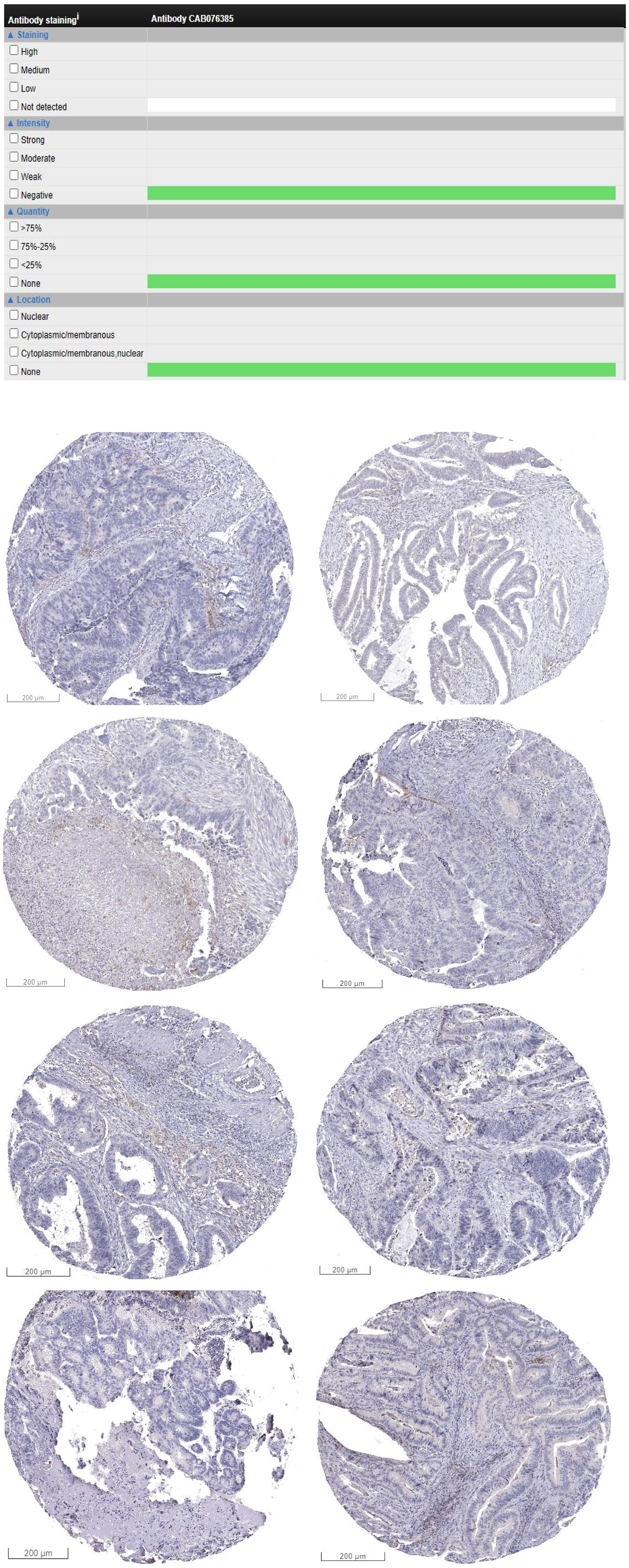


**B**

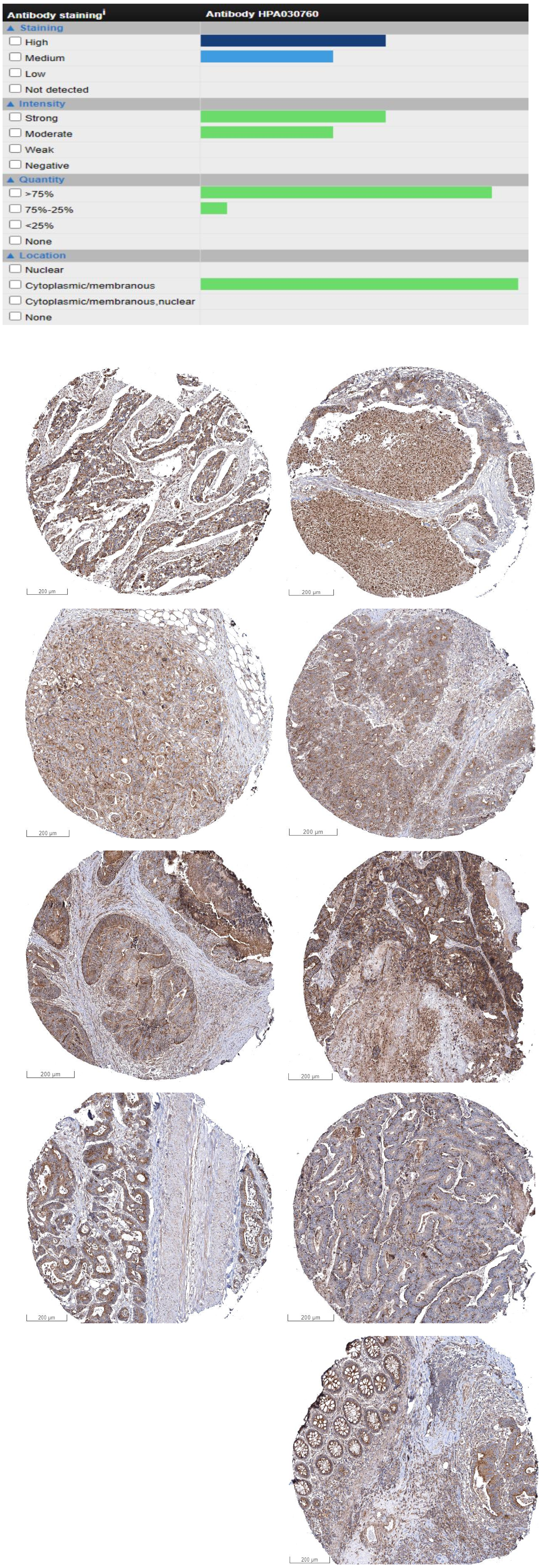

**Supplementary Figure 3**

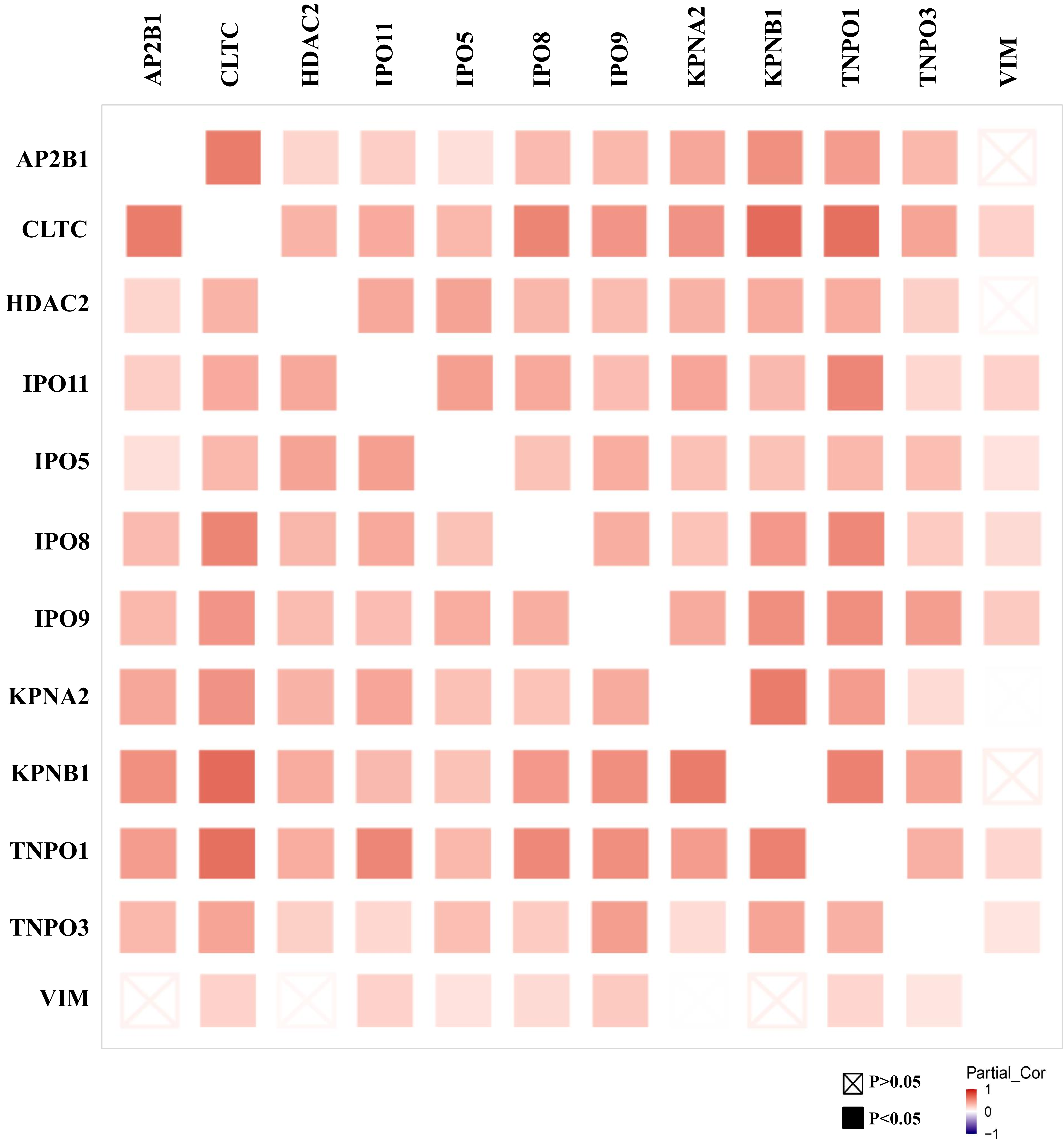

Supplement: Supplementary Figure 1 — Protein expression summary across cancers in the Human Protein Atlas (HPA) (A) PD-L1 (CD274) and (B) PSEN1 protein expressions in different type of TCGA tumors were summarized in the HPA. The color-coded bars indicate the percentages of the patients with high/medium PD-L1 or PSEN1 protein levels. Those with undetectable levels are shown in white bars. [file DataSheet_1.pdf]
